# Ambulation capacity, age, immunosuppression, and mechanical ventilation are risk factors of in-hospital death in severe COVID-19: a cohort study

**DOI:** 10.1016/j.clinsp.2022.100075

**Published:** 2022-07-04

**Authors:** Erika Christina Gouveia e Silva, Ana Carolina Basso Schmitt, Caroline Gil de Godoy, Amislaine Cristina Gambeta, Celso Ricardo Fernandes de Carvalho, Carolina Fu, Clarice Tanaka, Carlos Toufen Junior, Carlos Roberto Ribeiro de Carvalho, José Eduardo Pompeu

**Affiliations:** a Department of Physical Therapy, Speech Therapy, and Occupational Therapy, Faculdade de Medicina, Universidade de São Paulo, SP, Brazil; b Divisão de Pneumologia, Instituto do Coração (InCor), Faculdade de Medicina FMUSP, Universidade de Sao Paulo, São Paulo, SP, Brazil

**Keywords:** COVID-19, Adult, Older Adult, Hospitalisation, Ambulation Capacity

## Abstract

**Importance:**

Despite ambulation capacity being associated with a decreased level of physical activity and survival may be influenced by the functional capacity, studies have not addressed the association between ambulation capacity and death in patients hospitalized by COVID-19.

**Objective:**

To verify the functional, clinical, and sociodemographic risk factors associated with in-hospital death in individuals with severe COVID-19.

**Methods:**

It is a cohort retrospective study performed at a large tertiary hospital. Patients 18 years of age or more, of both sexes, hospitalized due to severe COVID-19 were included. Cases with dubious medical records and/or missing essential data were excluded. Patients were classified according to their ambulation capacity before the COVID-19 infection. Information regarding sociodemographic characteristics, in-hospital death, total hospital stays, Intensive Care Unit (ICU) stays, and the necessity of Mechanical Ventilation (MV) were collected from medical records and registered in a RedCap database. Multiple logistic regression analysis was used to identify possible factors associated with the in-hospital death rate.

**Results:**

Data from 1110 participants were included in the statistical analysis. The median age of the patients was 57 (46‒66) years, 58.42% (n = 590) were male, and 61.73% (n = 602) were brown or black. The case fatality rate during hospitalization was 36.0% (n = 363). In-hospital death was associated with ambulation capacity; dependent ambulators (OR = 2.3; CI 95% = 1.2–4.4) and non-functional ambulation (OR = 1.9; CI 95% = 1.1–3.3), age [older adults (OR = 3.0; CI 95% = 1.9‒4.), ICU stays (OR = 1.4; CI 95% = 1.2‒1.4), immunosuppression (OR = 5.5 CI 95% = 2.3‒13.5) and mechanical ventilation (OR = 27.5; CI 95% = 12.0–62.9).

**Conclusion and relevance:**

Decreased ambulation capacity, age, length of ICU stay, immunosuppression, and mechanical ventilation was associated with a high risk of in-hospital death due to COVID-19.

## HIGHLIGHTS

• Ambulation capacity, race, age, and immunosuppression increase the risk of in-hospital death. How might it impact clinical practice in the future?
• People with ambulation disabilities should be treated more carefully when they present the first symptoms of COVID-19, due to the high risk of developing a worse prognosis.
• Pandemic control recommendations should include the population with ambulation disability as a priority group in vaccination against COVID-19

## Introduction

Since January 2020, coronavirus COVID-19, caused by the SARS-CoV-2 virus, has affected more than 117 million people and caused the deaths of more than 2.6 million individuals worldwide.^1^ There is a variable in-hospital case fatality rate of COVID-19, ranging from 13% in hospitalized patients up to 37% in patients admitted to an Intensive Care Unit (ICU). In Brazil, the case fatality rate ranges from 31.8% in week 10% to 18.2% in week 40.^2^

Immunosuppression, obesity, diabetes, hypertension, heart disease, age over 65 years old, masculine sex, and smoking are factors associated with a more critical condition or death due to COVID-19.^3^ Initial clinical manifestations such as fever, shortness of breath, or dyspnea may also impair the progression of COVID-19.^3^ Race is another predictor of lethality and black people have higher mortality due to COVID-19 than white people (OR = 1.6; CI 95% = 1.2–2.0).^4^ Current epidemiological data on COVID-19 suggests that brown and black people may be more susceptible to SARS-CoV-2 infections.^5^ In Brazil, the death rates due to COVID-19 in brown and black people are three times higher than in white people.^6^ This higher susceptibility and the worse outcome could be related to the higher prevalence of diabetes, hypertension, obesity, asthma, and heart disease in brown and black people.^5^

Functional capacity, cardiovascular disease, and pneumonia are associated with a higher chance of death.^7^ A low level of physical activity has been associated with higher mortality in older adults.^7^ In heart failure patients, decreased physical activity is associated with poor functional physical performance and with an increased risk of hospitalization and mortality.^8^ Patients with COVID-19 with a low level of physical activity have a higher risk for hospitalization and death.^9^, ^10^, ^11^, ^12^ However, to our knowledge, no study has addressed the association between ambulation capacity and death in patients hospitalized by COVID-19.

Therefore, this study aimed to verify the functional, clinical, and sociodemographic risk factors associated with in-hospital death, and to compare the clinical and sociodemographic characteristics of individuals with severe COVID-19, according to their ambulation capacity prior to the disease.

The authors hypothesized that decreased ambulation capacity, and other previously reported clinical and sociodemographic characteristics, would be a factor associated with a higher risk of in-hospital death due to COVID-19 and, the participants with ambulation disability may present worse health conditions with a higher number of clinical and sociodemographic risk factors for in-hospital death.

## Methods

This retrospective cohort study was approved by the Ethics Committee of the Hospital das Clinicas of the Medical School of the University of São Paulo (number 4.052.246) and follows the Strengthening the Reporting of Observational Studies in Epidemiology (STROBE) reporting guideline.

### Study design and participants

This study included data from patients admitted to a referral public hospital for SARS-CoV-2 in São Paulo, Brazil, during the first (04/01/2020 to 11/30/2020) and second (01/01/2021 to 05/31/2021) waves of the COVID-19 pandemic. The authors included patients aged 18 years or older, of both sexes, and confirmed diagnosis of COVID-19 by Reverse Transcription-Polymerase Chain Reaction (RT-PCR). Cases with dubious information in the medical records and/or missing essential data were excluded.

Sociodemographic information (age, sex, and race), in-hospital death due to SARS-CoV-2, number of previous comorbidities, habits (alcohol consumption and smoking), hospital and ICU stays, Mechanical Ventilation (MV), and ambulation capacity before infection by SARS-CoV-2 was collected from medical records and registered in a RedCap database.

Participants were classified according to their ambulation capacity prior to infection by SARS-CoV-2 as independent, dependent, or non-functional ambulators. Individuals who could ambulate independently on non-level and level surfaces, stairs, and inclines were classified as independent ambulators. Individuals requiring continuous or intermittent light touch to assist balance or coordination from one or more persons to ambulate on level surfaces to prevent falls were classified as dependent ambulators. Individuals who were unable to walk or needed help were classified as non-functional ambulators. Ambulation classification was based on and adapted from a previous study.^13^

### Statistical analysis

Statistical analysis was performed using JASP (0.14.1) and Stata 14.4 (StataCorp, College Station, TX, USA) software. Kruskal Wallis, Mann-Whitney, and chi-squared tests were performed to analyze the association between demographic and clinical characteristics with in-hospital death and to compare the characteristics of the groups. The post hoc test of Dunn was applied to paired comparisons. The case fatality rate was estimated per point and 95% Confidence Interval. The multiple logistic regression model was applied, estimating the net weight of possible demographic and clinical factors associated with in-hospital death after bivariate analysis (p < 0.20), and adjusted by age and wave. In all statistical tests, the level of significance was previously established at α = 0.05 and the Confidence Interval (CI) was set at 95%.

## Results

A total of 1011 patients were included in this study, the median age of 57 (46‒66) years, 58.4% (n = 590) male, and 61.7% (n = 602) brown or black. The most prevalent comorbidities were diabetes mellitus (28.4%; n = 287), and obesity (24.4%; n = 246). The median hospital stay was 16 (10‒28) days, and the median ICU stay was 12 (6‒21) days. In-hospital case fatality rate was 36.0% (n = 363) (CI 95% = 33.0–39.0). Table 1 shows the demographic and clinical characteristics of patients according to their ambulation capacity before infection by SARS-CoV-2.

**Table 1:** Patient demographic and clinical characteristics by ambulation capacity before hospitalisation.

	Independent ambulator	Dependent ambulator	Non-Functional ambulator	Total	p-value
	n = 686 (67.85%)	n = 119 (11.77%)	n = 206 (20.38%)	(n = 1011)	
**Age**					0.012^KW^
Median (Q1, Q3)	57 (45‒66)	62 (50‒71)	57 (47‒65)	57 (46‒66)	
**Wave n (%)**					<0.001^X2^
First Wave	381 (74.56)	83 (16.24)	47 (9.20)	511 (50.59)	
Second Wave	305 (61.12)	36 (7.21)	158 (31.67)	499 (49.41)	
**Age group n (%)**					0.013^KW^
Adults	394 (69.49)	52 (9.17)	121 (21.34)	567 (56.14)	
Older Adults	292 (65.91)	67 (15.12)	84 (19.97)	443 (43.86)	
**Sexes n (%)**					0.681^X2^
Female	279 (66.43)	51 (12.14)	90 (21.43)	420 (41.58)	
Male	407 (68.98)	68 (11.53)	115 (19.49)	590 (58.42)	
**Race n (%)**					<0.001^X2^
White	230 (60.37)	37 (9.71)	114 (29.92)	377 (38.27)	
Brown/Black	437 (72.35)	79 (13.08)	88 (14.57)	602 (61.73)	
**Habits n (%)**					
Smoking	96 (72.73)	24 (18.18)	12 (9.09)	132 (13.39)	<0.001^X2^
Alcoholism	20 (76.92)	3 (11.54)	3 (11.54)	26 (2.64)	0.483^X2^
**Number of comorbidities, n (%)**					0.001^KW^
Median (Q1, Q3)	1 (0‒3)	2 (0.7‒3)	1 (0‒2)	1 (0‒3)	
**Comorbidities n (%)**					
Cardiovascular disease	106 (70.20)	19 (12.58)	26 (17.22)	151 (14.95)	0.588^X2^
COPD	40 (70.18)	7 (12.28)	10 (17.54)	57 (5.64)	0.868^X2^
Renal disease	12 (80.00)	2 (13.33)	1 (6.67)	15 (1.49)	0.416^X2^
Systemic arterial hypertension	254 (67.73)	50 (13.33)	71 (18.94)	375 (37.13)	0.0413^X2^
Diabetes Mellitus	199 (69.34)	44 (15.33)	44 (15.33)	287 (28.42)	0.010^X2^
Obesity	166 (67.21)	29 (11.74)	52 (21.05)	246 (24.36)	0.943^X2^
Hepatic disease	7 (57.85)	5 (38.46)	1 (7.69)	13 (1.29)	0.009^X2^
Hematological disease	9 (81.82)	2 (18.18)	0 (0.00)	11 (1.09)	0.228^X2^
Dyslipidemia	75 (71.43)	13 (12.38)	17 (16.19)	106 (10.50)	0.543^X2^
Neurological disease	14 (48.28)	7 (24.14)	8 (27.58)	29 (2.87)	0.042^X2^
Immunosuppression	50 (72.46)	11 (15.94)	8 (11.60)	69 (6.83)	0.130^X2^
**Length of hospital stay (days)**					0.017^KW^
Median (Q1, Q3)	16 (10‒27)	21 (12‒32)	15 (8‒26)	16 (10‒28)	
**Length of ICU stay (days)**					0.020^KW^
Median (Q1, Q3)	11 (6‒20.25)	13 (7‒23.50)	12 (7‒21)	12 (6‒21)	
**VM n (%)**					0.006^KW^
Adults	223 (63.35)	40 (11.36)	89 (25.29)	352 (53.50)	
Older Adults	201 (65.69)	45 (14.71)	60 (19.60)	306 (46.50)	
**Death n (%)**					<0.001^KW^
Adults	86 (56.21)	21 (13.73)	46 (30.06)	153 (42.15)	
Older Adults	128 (60.95)	33 (15.71)	49 (23.34)	210 (57.85)	

Data are expressed as median and or interquartile (Q1 and Q3); number of patients and percentage; n (%), Percentage of patients; ICU, intensive care unit; VM, Mechanical Ventilation; KW, Kruskal Wallis test; X^2^, Chi-Squared test.

**Table 2:** Demographic and clinical characteristics of in-hospital deaths among patients with COVID-19.

	Death		p-value
	Yes (n = 364)	No (n = 647)	
**Age**			<0.001^MW^
Median (Q1, Q3)	62 (51‒70)	55 (44‒63)	
**Age group n (%)**			<0.001^MW^
Adults	154 (27.11)	414 (72.89)	
Older Adults	210 (47.40)	233 (52.60)	
**Sexes n (%)**			<0.001^X2^
Female	110 (26.13)	311 (73.87)	
Male	254 (43.05)	336 (56.95)	
**Race n (%)**			<0.001^X2^
White	103 (27.03)	278 (72.97)	
Brown / Black	259 (42.80)	346 (57.20)	
**Wave n (%)**			<0.001^X2^
First wave	220 (43.05)	291 (56.95)	
Second wave	144 (28.80)	356 (71.20)	
**Habits n (%)**			
Smoking	62 (46.97)	70 (53.03)	0.010^X2^
Alcoholism	13 (50.00)	13 (50.00)	0.160^X2^
**Number of comorbidities n (%)**			0.100^X2^
Median (Q1, Q3)	2 (0‒3)	1 (0‒2)	
**Comorbidities n (%)**			
Cardiovascular disease	50 (33.11)	101 (66.89)	0.422^X2^
COPD	19 (33.33)	38 (66.67)	0.665^X2^
Renal disease	8 (53.33)	7 (46.67)	0.159^X2^
Arterial hypertension	146 (38.93)	229 (61.07)	0.136^X2^
Diabetes Mellitus	103 (35.89)	184 (64.11)	0.962^X2^
Obesity	71 (28.63)	177 (71.37)	0.005^X2^
Dyslipidaemia	48 (45.71)	57 (54.29)	0.029^X2^
Hepatic disease	6 (46.15)	7 (53.85)	0.443^X2^
Neurologic disease	11 (37.93)	18 (62.07)	0.826^X2^
Haematological disease	3 (27.27)	8 (72.73)	0.544^X2^
Immunosuppression	39 (56.52)	30 (43.48)	<0.001^X2^
**Length of hospital stay (days)**			0.004^MW^
Median (Q1, Q3)	18 (10‒30)	15 (9‒24.25)	
**Length of ICU stay (days)**			<0.001^MW^
Median (Q1, Q3)	10 (4‒19)	14 (8‒23)	
**VM n (%)**			<0.001^X2^
Yes	347 (52.74)	311 (47.27)	
No	17 (4.83)	335 (95.17)	
**Ambulation Capacity n (%)**			<0.001^MW^
Independent ambulator	214 (31.19)	472 (68.81)	
Dependent ambulator	54 (45.38)	65 (54.62)	
Non-Functional ambulator	95 (46.34)	110 (53.66)	

Data are expressed as median and interquartile (Q1 and Q3) or number of patients and percentage; n (%), Percentage of patients; ICU, Intensive Care Unit; VM, Mechanical ventilation; MW, Mann-Whitney *U* test; X2, Chi-Squared test; COPD, Chronic Obstructive Pulmonary Disease.

**Table 3:** Logistic regression model adjusted for in-hospital deaths.

Demographic and clinical characteristics		Death	
		OR (95% IC)	p-value
**Older Adult**		3.0 (1.9‒4.7)	<0.001
**Male**		2.5 (1.6‒3.8)	<0.001
**Brown / Black**		1.8 (1.1‒3.1)	0.028
**Immunosuppression**		5.5 (2.3‒13.5)	<0.001
**Length of hospital stay (days)**		0.7 (0.6‒0.7)	<0.001
**Length of ICU stay (days)**		1.4 (1.2‒1.4)	<0.001
**MV**		27.5 (12.0–62.9)	<0.001
**Ambulator**	Independent	Ref	
	Dependent	2.3 (1.2–4.4)	0.014
	Non-Functional	1.9 (1.1–3.3)	0.032
**Second Wave**		1.3 (0.7–2.2)	0.402

MV, Mechanical Ventilation; Ref, Reference; OR, Odds Ratio; 95% IC, Confidence Interval 95%; p, significance.

### Ambulation capacity and comorbidities (Table 1)

Participants were classified as independent ambulators (67.9%; n = 686), dependent ambulators (11.7%; n = 119) and non-functional ambulators (20.4%; n = 206).

There was a significant association between ambulation capacity before COVID-19 with the prevalence of diabetes *mellitus* (*X*^2^ = 9.28, p = 0.014), hepatic disease (*X^2^* = 9.37, p = 0.022), and neurological disease (*X^2^* = 6.35, p = 0.042).

### Ambulation capacity and Mechanical ventilation (Table 1)

The need for MV (H^2^ = 10.377, p = 0.006) to treat COVID-19 was greater in the dependent and non-functional ambulator groups than for independent ambulators (Dunn post hoc test, p = 0.022 and p = 0.002, respectively). There was no significant difference between dependent versus non-functional ambulators (Dunn post hoc test, p *=* 0.410).

### Ambulation capacity and in-hospital death (Table 1)

The ambulation capacity before COVID-19 affected in-hospital death (H^2^ = 20.924, p < 0.001), being higher in the dependent and non-functional ambulator groups than for independent ambulators (Dunn post hoc test, p = 0.001 and p < 0.001, respectively). There was no significant difference between dependent versus non-functional ambulators (p = 0.431).

### Ambulation capacity and clinical and sociodemographic characteristics according to outcome (in-hospital death or discharge) (Table 2)

There was a significant association between the COVID-19 outcome (in-hospital death or discharge) and ambulation capacity before hospitalization (*X*^2^ = 20.95, p < 0.001). COVID-19 outcome was also associated with sex (*X^2^* = 30.54, p < 0.001), race (*X^2^* = 25.04, p < 0.001), COVID-19 wave (*X*^2^ = 22.28, p < 0.001), MV (*X*^2^ = 228.32, p < 0.001), smoking (*X^2^* = 6.67, p = 0.010), obesity (*X*^2^ = 7.76, p = 0.044), dyslipidemia (*X*^2^ = 4.80, p = 0.029), and immunosuppression (*X*^2^ = 13.53, p < 0.001).

The authors compared the characteristics of those participants who died during hospitalization (in-hospital death group) with those who were discharged from the hospital (discharge group). There were significant differences between the in-hospital mortality group and the discharge group regarding (1) age [in-hospital death group (median = 62 years); discharge group (median = 55 years); (W = 88328, *Hodges-Lehmann estimate* = -6, *Rank-Biserial Correlation* = 0.250, p < 0.001)]; (2) length of hospital stay [in-hospital death group (median = 15 days); discharge group (median = 18 days); (W = 130302, *Hodges-Lehmann estimate* = -2, *Rank-Biserial Correlation* = -0.108, p = 0.004)]; length of ICU stay [in-hospital death group (median = 14 days); discharge group (median = 10 days); (W = 73453, *Hodges-Lehmann estimate* = -4, *Rank-Biserial Correlation* = -0.217 p < 0.001)].

### Predictors of in-hospital death (Table 3)

Logistic regression adjusted for in-hospital death showed an association between ambulation capacity (dependent ambulator OR = 2.3; CI 95% = 1.2–4.4 and non-functional ambulator OR = 1.9; CI 95% = 1.1–3.3). In-hospital death was also associated with older adults (OR = 3.0; CI 95% = 1.9–4.7), females (OR = 2.5; CI 95% = 1.6–3.8), brown or black race (OR = 1.8; CI 95% = 1.1–3.1), immunosuppression (OR = 5.5; CI 95% = 2.3–13.5), length of hospital stay (OR = 0.7; CI 95% = 0.6–0.7), length of ICU stay (OR = 1.4; CI 95% = 1.2–1.4) and MV (OR = 27.5; CI 95% = 12.0–62.9).

## Discussion

To our knowledge, this is the first study that investigated if ambulation capacity is a risk factor associated with the outcome of patients hospitalized with severe COVID-19. The main finding of the present study was that ambulation capacity before COVID-19 is a strong predictor for in-hospital death. People with worse functional capacity, mainly older adults, suffer a higher impact of hospitalization due to several diseases.^16^, ^17^, ^18^ Specifically, in patients with COVID-19, the lower level of physical activity was a risk factor for worse outcomes, including death.^10^ Physical activity seems to play a protective role against COVID-19 even at levels below those currently recommended, probably due to an anti-inflammatory action and positive effects on the immune system.^16^, ^17^, ^19^

Another important finding of the present study was that race (brown and black) is associated with in-hospital death in patients with COVID-19. Brown and black patients present higher case fatality rates (42.80%) compared to white patients (27.03%). The present results corroborate the findings of Price-Haywood et al. 2020^20^ and Karmakar et al. 2021^21^ in which black and brown individuals presented a higher risk of worse prognosis and death by COVID-19. Additionally, Araujo et al. 2020^6^ reported that hospitalizations amongst black and brown people increased throughout the pandemic in Brazil. The mechanism for higher lethality of black people can be explained by their lower and slower excretion of sodium, which results in the suppression of the Aninnine-Aldosterone-Angiotensin System (AAR), thereby tending towards sodium retention and arterial hypertension.^22^ Individuals with hypertension express more Aangiotensin-Converter Enzyme (ACE-2), the main receptor used by SARS-CoV-2 to invade host cells. The link between the virus and ACE-2 increases the levels of angiotensin type II causing tissue injury and dysregulation of the aninnine-angiotensin-aldosterone system.^23^

The present results show that obesity, dyslipidemia, and immunosuppression are risk factors for mortality. Obese individuals with COVID-19 were more severely affected and had a worse prognosis,^24^ as well as an increased risk of death.^25^ Probably, obese individuals suffer an additional reduction in lung volume and capacity and present increased airway resistance.^24^^,^^25^ Dyslipidemia was present in 13% of the present study's participants. A study observed that dyslipidemia is associated with a 60% increase in the risk of short-term mortality.^26^ Individuals with dyslipidemia infected with SARS-CoV-2 are more likely to have acute cardiovascular events and progress to serious disease,^27^ probably due to prior vascular damage caused by dyslipidemia.^26^ The present results corroborate a systematic review and meta-analysis^9^ that showed that MV, age (older adults), length of stay in ICU, and immunosuppression are strong predictors of in-hospital death in patients with severe COVID-9.

In the present study, the in-hospital case fatality rate (36%) was higher than previously reported (20% to 31%).^14^^,^^15^^,^^28^ The higher case fatality rate of the present study could be related to the data collection site which was a reference hospital for severe and moderate cases of COVID-19, where only those patients with significant clinical worsening were transferred, often already being admitted for invasive MV.

Immunosuppression was a prognostic factor for death. An adequate immune response for the creation of antibodies is important for protection against COVID-19 infection, and several studies have demonstrated a decreased serum antibody response to SARS-CoV-2 in severe cases of ICU patients.^7^^,^^9^ Individuals with deficient immune responses are more susceptible to diseases, particularly certain respiratory diseases, such as influenza, as well as immune responses harmful to vaccination.^27^

Length of ICU stay (OR = 1.4; CI 95% = 1.2‒1.4) and MV (OR = 27.5; CI 95% = 12.0–62.9) were strong predictors of in-hospital death. The length of hospital stay depends on the severity of the condition and the need for care support, ranging from the use of oxygen therapy to ICU admission.^29^^,^^30^ Another explanation for this strong risk factor due to MV was the fact that most of these patients developed severe acute respiratory failure and therefore had to be intubated. This intervention was necessary because in most cases, there was the development of Acute Respiratory Distress Syndrome (ARDS), a serious condition that can lead to death. The necessity of MV is related to the severity of symptoms that leads to longer stays in the ICU. It is notable that the present study's patients presented severe symptoms and a greater propensity to needing MV. Additionally, individuals who survived a long ICU stay may develop post-intensive care syndrome that affects their physical, cognitive, and mental functions. Its main causes are interventional, environmental, and psychological factors, including the need for MV.^30^ This syndrome can negatively influence the quality of life of the individual, and long-term follow-up studies on persistent symptoms in patients after hospital discharge following COVID-19 are considered fundamental.^31^ In addition, hospitalization can lead to disabilities due to underlying disease and low levels of mobility, even with adequate treatment. This condition can cause serious long-term consequences, including an increased risk of hospital readmission, mortality, and symptoms possibly lasting for months after hospital discharge.^32^

The present results showed a higher risk of death in older adults (OR = 3.0; CI 95% = 1.9‒4.7). COVID-19 affects all age groups, but its severity increases for groups above 50 years, probably due to physiological changes resulting from the aging process and the presence of a greater number of comorbidities.^27^^,^^28^ Due to SARS-CoV-2 viral infection, pro-inflammatory cytokines are stimulated in response to changes in the hematogenous pathway causing a prothrombotic effect, which in turn stimulates the proliferation of immune cells responsible for fighting chronic inflammations. Especially in older adults, due to the immunosenescence, tissue damage with oxygenation deficiency can cause important tissue distress and worse evolution of COVID-19.^27^ However, age is not a risk factor for ICU admission, nor a determinant for the use of MV. The present results demonstrated that adults were the group who most needed MV as a therapeutic option (53.50%; n = 352), according to a previous study.^13^^,^^33^ that showed that the risk factors for death in individuals submitted to MV for COVID-19 are the severity of the condition and functional disability.^14^^,^^15^

In agreement with previous studies,^5^^,^^34^ the present results showed that men presented a higher risk of death from COVID-19 (OR = 2.5; CI 95% = 1.6‒3.8). The higher susceptibility of men to acquiring viral infections^5^^,^^35^ and the difference in testosterone and estrogen levels between men and women may predispose males to more frequently severe cases of COVID-19.^5^ Additionally, men are less engaged with their health care, especially for chronic diseases, which can be associated with greater morbidity and more complications due to COVID-19.^5^^,^^34^ However, following the elimination of viral infection, women maintain high immunological responses, which may increase the risk of post-infection complications,^5^ and more long-term symptoms than men.^34^^,^^35^

The present study corroborates the findings of previous studies regarding clinical and sociodemographic risk factors for in-hospital death in severe COVID-19 and advances our knowledge by demonstrating the association between prior ambulation capacity and worse prognostic outcomes in severe COVID-19 hospitalized patients.

The authors suggest that the ambulation capacity factor be assessed at hospital admission. Moreover, there is a consensus regarding the need to encourage physical activity, especially for the population with some ambulation impairment, to prevent several negative health outcomes. The present results reinforce the need to include people with functional deficiency in the priority vaccination groups and special attention during hospitalization due to the high risk of worse outcomes from COVID-19.

### Strengths and limitations

The present study has the following strengths:

The present sample was composed of SARS-CoV-2 patients hospitalized in a referral hospital for critically ill patients in the city of São Paulo, Brazil.

No other study has analyzed ambulation capacity before hospitalization as a predictor of prognosis and death in COVID-19.

The present results need to be interpreted considering the following limitations:

The population being studied was drawn from a hospitalized sample of critically ill patients. Hence, the results are not generalizable to light or moderate COVID-19 cases.

Because of the selected sample, some parts of the analysis may suffer from selection bias and effects, such as collider and missing bias.

The authors did not collect data regarding the specific type of immunosuppression and thus, our results could not ascertain the risk for each specific type.

## Conclusion

Decreased ambulation capacity, age, race, length of ICU stay, immunosuppression, and mechanical ventilation were associated with a high risk of in-hospital death due to COVID-19.

## Ethical approval

This study was approved by the Ethics Committee of the Hospital das Clinicas of the Medical School of the University of São Paulo, Brazil (4.052.246) Certificate of Presentation for Ethical Appreciation.

## Authors’ contributions

There is no conflict of interest on the part of the authors.

## CRediT authorship contribution statement

**Erika Christina Gouveia e Silva:** Methodology, Validation, Formal analysis, Investigation, Resources, Data curation, Writing – original draft, Writing – review & editing, Project administration. **Ana Carolina Basso Schmitt:** Methodology, Validation, Investigation, Resources, Data curation, Writing – original draft, Writing – review & editing, Project administration. **Caroline Gil de Godoy:** Methodology, Validation, Resources, Data curation, Investigation, Writing – review & editing. **Amislaine Cristina Gambeta:** Validation, Resources, Writing – review & editing. **Celso Ricardo Fernandes de Carvalho:** Validation, Resources, Writing – review & editing. **Carolina Fu:** Validation, Resources, Writing – review & editing. **Clarice Tanaka:** . **Carlos Toufen Junior:** . **Carlos Roberto Ribeiro de Carvalho:** . **José Eduardo Pompeu:** Conceptualization, Methodology, Validation, Formal analysis, Investigation, Resources, Writing – review & editing, Project administration.

## Declaration of Competing Interest

The authors declare no conflicts of interest.
